# Myofibroblast-Derived Exosome Induce Cardiac Endothelial Cell Dysfunction

**DOI:** 10.3389/fcvm.2021.676267

**Published:** 2021-04-23

**Authors:** Prabhat Ranjan, Rajesh Kumari, Sumanta Kumar Goswami, Jing Li, Harish Pal, Zainab Suleiman, Zhongjian Cheng, Prasanna Krishnamurthy, Raj Kishore, Suresh Kumar Verma

**Affiliations:** ^1^Division of Cardiovascular Disease, Department of Medicine, The University of Alabama at Birmingham, Birmingham, AL, United States; ^2^Molecular and Cellular Pathology, Department of Pathology, The University of Alabama at Birmingham, Birmingham, AL, United States; ^3^Department of Biomedical Engineering, The University of Alabama at Birmingham, Birmingham, AL, United States; ^4^Center for Translational Medicine, Temple University, Philadelphia, PA, United States

**Keywords:** cardiac fibrosis, angiogenesis, microRNA, exosome (vesicle), endothelial (dys)function

## Abstract

**Background:** Endothelial cells (ECs) play a critical role in the maintenance of vascular homeostasis and in heart function. It was shown that activated fibroblast-derived exosomes impair cardiomyocyte function in hypertrophic heart, but their effect on ECs is not yet clear. Thus, we hypothesized that activated cardiac fibroblast-derived exosomes (FB-Exo) mediate EC dysfunction, and therefore modulation of FB-exosomal contents may improve endothelial function.

**Methods and Results:** Exosomes were isolated from cardiac fibroblast (FB)-conditioned media and characterized by nanoparticle tracking analysis and electron microscopy. ECs were isolated from mouse heart. ECs were treated with exosomes isolated from FB-conditioned media, following FB culture with TGF-β1 (TGF-β1-FB-Exo) or PBS (control) treatment. TGF-β1 significantly activated fibroblasts as shown by increase in collagen type1 α1 (*COL1*α*1*), periostin (*POSTN*), and fibronectin (*FN1*) gene expression and increase in Smad2/3 and p38 phosphorylation. Impaired endothelial cell function (as characterized by a decrease in tube formation and cell migration along with reduced *VEGF-A, Hif1*α*, CD31*, and *angiopoietin1* gene expression) was observed in TGF-β1-FB-Exo treated cells. Furthermore, TGF-β1-FB-Exo treated ECs showed reduced cell proliferation and increased apoptosis as compared to control cells. TGF-β1-FB-Exo cargo analysis revealed an alteration in fibrosis-associated miRNAs, including a significant increase in miR-200a-3p level. Interestingly, miR-200a-3p inhibition in activated FBs, alleviated TGF-β1-FB-Exo-mediated endothelial dysfunction.

**Conclusions:** Taken together, this study demonstrates an important role of miR-200a-3p enriched within activated fibroblast-derived exosomes on endothelial cell biology and function.

## Introduction

The endothelium maintains normal vascular tone and blood fluidity under normal homeostatic conditions. Increased vascular tone, hypertrophic remodeling, and inflammation are associated with cardiovascular diseases, including heart failure (1–6). In heart, the intercellular interaction of cardiomyocytes (CM) and non-myocyte cells are mainly responsible for the initiation and maintenance of functional homeostasis (7, 8). Transforming growth factor beta-1 (TGF-β1), a profibrotic cytokine, plays an important role in profibrotic signaling, and fibrosis in the heart (9, 10). It enhances production of extracellular matrix proteins by fibroblasts. Furthermore, as fibrosis progresses, it leads to tissue damage and inflammation (11). In this process, endothelial cells lose their cellular integrity and undergo endothelial to mesenchymal transition (EndMT) (12, 13). Buckley et al. have reported that stromal fibroblasts can also modify the function of endothelial cells (14). However, the underlying mechanisms of fibroblast-endothelial cell communication are not yet fully understood.

Recent studies, including those from our group, suggest that a variety of cells, including cardiac cells, secrete exosomes as potent paracrine effectors and that exosomes largely recapitulate the reparative/pathological properties of the parent cell in cardiac repair/disease processes (15, 16). Exosomes may mirror their parent cell's molecular profile in response to physiological and pathological stimuli. Signaling molecules packaged within the exosomes can modulate many cellular processes, including survival, proliferation, differentiation, and commitment, of the target/recipient cells (17, 18). MicroRNAs (miRNAs) have emerged as regulators of cell-cell communication and as paracrine signaling mediators during physiological and pathological processes in heart and other organs (19, 20). The miRNAs are short, non-coding nucleotides regulating expression of target genes by mRNA degradation or translational repression (21). Alterations in miRNAs associated with dysfunctional gene are observed in many cardiovascular diseases (22). Interestingly, evaluation of the contents of cardiac fibroblast–derived exosomes revealed a relatively high abundance of many miRNAs (22). Roles for miRNA-dependent and vesicle-mediated communication between endothelial cells and other cardiovascular cells are gradually being revealed (20, 23). However, it is unclear whether activated fibroblasts/myofibroblasts regulate endothelial cell function via exosomes. Therefore, we hypothesized that myofibroblasts secrete exosomes packed with antiangiogenic/profibrotic factors, which impede endothelial cell function.

In this study, we identified that activated fibroblasts secrete altered exosomes (TGF-β1-FB-Exo) with upregulated miR-200a-3p as a potent paracrine molecule that induces cardiac endothelial cell dysfunction. We revealed that inhibition of miR-200a-3p in fibroblast-derived exosomal cargo rescued functional properties of endothelial cells. Therefore, our observation can facilitate design of interventions that can prevent endothelial dysfunction and associated cardiovascular diseases.

## Materials and Methods

### Cell Isolation and Culture (NRVF and Endothelial Cell)

Neonatal rat ventricular fibroblasts (NRVF) were isolated from the hearts of 1–2-day-old Sprague Dawley rat pups (Charles River, PO Box 31346, Hartford, CT, USA) according to a standard protocol as previously described (24). Nonmyocyte NRVF cells were separated using a discontinuous Percoll gradient method. NRVF were grown to confluence and subsequently trypsinized and plated on 100 mm tissue culture dishes (first passage) containing DMEM media with 4.5 g/L glucose (Corning, Catalog #10-017-CV), 10% fetal bovine serum (Thermofisher Scientific, Catalog #11-965-118), and 1% penicillin/streptomycin and maintained at 37°C in humid air with 5% CO_2_. At ~70% confluency, the culture media was changed to serum-free DMEM and experiments were performed.

Mouse endothelial cell isolation and culture were performed with little modification as described previously (25). Briefly, 6–8-week-old C57BL/6J mice (Jackson Labs, P.O. Box 90260, Chicago, IL, USA) mice hearts were excised and digested in collagenase type I (1 mg/ml, Sigma-Aldrich, Catalog #C5894) at 37°C for 25–30 min. After digestion, cells were filtered through a double layer 40 μm nylon mesh and centrifuged (400 g, 4°C). Following two series of washing with DMEM, cells were incubated with sheep anti-rat magnetic beads pre-coated with purified rat anti-mouse CD31 (BD Pharmingen, Catalog #553370) at 4°C for 15 min for positive selection. After affinity binding, magnetic beads were washed 6 times with DMEM containing 10% FBS. Finally, purified cells were plated on 1% bovine gelatin (Sigma, Catalog #G9391)-coated plate in endothelial cell growth medium (EBM-2 MV culture medium with growth factors). Media were changed on the next day and every other day during the experiments. Endothelial cells in passage 2–3 were used in most experiments.

### NRVF Treatment, Exosome Isolation, and Characterization

NRVFs were either treated with bovine serum albumin (BSA) or 5 ng/ml TGF-β1 (R&D System, Catalog #7666-MB-005) at about 70% confluency in serum-free medium. After 48 h, exosomes were isolated from NRVF-conditioned media (26). In brief, conditioned media collected from NRVF culture was centrifuged at 10,000 g for 20 min at 4°C to remove cells and cellular debris. Filtrate was subjected to ultracentrifugation at 100,000 g for 1.5 h at 4°C. This step was repeated by resuspending exosome pellet in sterile PBS. Finally, exosome pellets were resuspended in 50 μl PBS and stored at −80°C till further use. Nanoparticle tracking analysis (NanoSight NS300, Malvern Panalytical) was performed to determine exosome size and quantity. The size and shape of exosomes were further measured by Tecnai Spirit T12 transmission electron microscopy (Thermofisher) in High Resolution Imaging Core Facility at The University of Alabama at Birmingham, Alabama.

### Endothelial Cell Treatment With Exosomes

Primary mouse cardiac endothelial cells (Passage 1) were seeded in 6-well-plates with EBM-2 MV media containing growth factors (Lonza, Catalog #CC-3202). At 70–80% confluency, ECs were treated with exosomes from PBS-, BSA (control)- or TGF-β1-treated FBs (TGF-β1-FB-Exo) (30,000 exosomal particles/ml) in EBM-2 MV basal medium with 2.5% exosome-depleted FBS for 48 hrs. Cells were trypsinized to perform assays, including migration, tube formation, MTT [3-(4.5-di**m**ethyl**t**hiazol-2-yl)-2.5-diphenyl**t**etrazolium bromide] and TUNEL. We excluded all the dead cells before seeding for tube formation and migration assay to avoid the angiogenic impairment due to cell death. Further, cells were harvested for biochemical (RNA and protein) analyses in their corresponding lysis buffer.

### Tube Formation Assay

Approximately 1.5 × 10^4^ mouse cardiac ECs (following exosome treatment) were seeded on Matrigel (Corning)-coated 48-well-plates (BD Falcon) as described previously (25). After 6–8 hrs., cells were observed and imaged under phase-contrast microscopy (Nikon Eclipse Inverted Phase Contrast Microscope, Spectra Services, Inc.; x100 magnification). The total branching lengths were measured in each well using Angiogenesis Analyzer (ImageJ 1.52 t; NIH, USA) and are represented as μm/field.

### Migration Assay

Migration of ECs toward a gradient of growth factors (EGM-2MV Microvascular Endothelial Cell Growth Medium-2, Lonza, Catalog #CC-4147) was performed in a 24-well Transwell Boyden Chamber (8.0 mm pore size, polycarbonate membrane, Corning Costar; Corning Incorporated Life Sciences, Acton, MA) as described previously (25). Briefly, 750 μl EBM-2 MV growth medium (Lonza, Catalog #CC-3202) with 10% exosome-depleted FBS were added to the lower compartment. ECs (1 × 10^5^ cells), pre-treated with exosomes, were added to the upper compartment (insert) in 200 μl of Opti-MEM (Thermo Fisher Scientific). After incubation at 37°C for 12–16 h, the cells that migrated through the membrane were stained with Giemsa stain (Sigma) and counted manually under phase contrast microscopy (Nikon Eclipse Inverted Phase Contrast Microscope, Spectra Services, Inc.; x100 magnification) in each well. Data from at least five biological replicates are expressed as the mean number of cells per well that migrated through the membrane.

### MTT Viability Assay

The MTT assay (Sigma, Cell Proliferation Kit I) was performed according to the manufacturer's instruction to assess endothelial cell viability after exosome treatments. Exosome-treated ECs were seeded in 96-well-plates. In all experiments, 20 μl of 5 mg/ml MTT reagents (3- [4.5-Dimethylthiazol-2-yl]-2.5-diphenyltetrazolium bromide, Sigma-Aldrich) were added to each well and plates were incubated for 3 h at 37°C. After washing with PBS, 150 μl of MTT solvent solution (4 mM HCl, and 0.1% Nonidet P-40 in isopropanol) were added to each well for solubilizing formazan. Absorbance of formazan was read, after 30 min, at 570 nm on EPOCH2 Microplate Reader (BioTek Instruments, Inc., Vermont, USA). The cell viability was considered directly proportional to the level of formazan produced.

### Terminal Deoxynucleotidyl Transferase-Mediated dUTP Nick End-Labeling (TUNEL) Staining Assay

Primary ECs were seeded on coverslips in a 24-well-plate precoated with gelatin. After 24 hrs., cells were treated with PBS or exosomes isolated from NRVFs treated with either BSA or TGF-β1. After 48 h. of the treatment, cells were washed with PBS and fixed with 4% paraformaldehyde (PFA). Apoptotic cells were determined by TUNEL staining as per manufacturer's instructions (Cell death detection assay; Roche, Indianapolis, IN). ECs were counter stained with CD31 antibodies before TUNEL staining. DAPI was used to stain the nuclei.

### RNA and Protein Isolation From Cells

Total RNA was isolated from cells (NRVFs and ECs) using RNeasy Mini Kit (Qiagen) and finally eluted in 50 μl of nuclease-free water as per the manufacturer's instructions and quantified by taking the ratio of OD_260/280_ on epoch 2 microplate spectrophotometer. For protein, cells were lysed in cell lysis buffer (Cell Signaling Technology, Catalog# 9803) supplemented with protease inhibitors and centrifuged at 14,000 rpm for 15 min at 4°C. The cell lysate was collected and stored at −80°C. Protein concentration was estimated by using BCA Protein Assay Kit (Thermo Fisher Scientific, USA).

### RNA Analysis for Fibrosis and Angiogenesis

We synthesized cDNA using high-capacity cDNA reverse transcription kit (Applied Biosystems) from total RNA. The gene expression of selected genes were measured using quantitative reverse transcription polymerase chain reaction (TaqMan RT-PCR; Applied Biosystems) technology. The TaqMan specific primer 18S small nucleolar RNA was used for normalizing the threshold delta-delta cycle method. Detailed information for each primer is provided in the (Supplementary Table 1).

### Immunostaining Assay

Primary fibroblasts were seeded in 4 well Nunc Lab-Tek Chamber Slide (Thermofisher, Catalog #177399) and cells were treated with BSA and TGF-β1. After treatment, cells were washed with PBS and fixed with 4% paraformaldehyde (PFA) and permeabilized with 0.2% Triton-X 100 in 1X PBS for 15 min. Further, cells were immune-stained with antibodies against α smooth muscle actin (αSMA monoclonal, Cell Signaling Technology, Catalog #19245) and Anti-Fibronectin antibody (Rabbit monoclonal, abcam, Catalog #ab206928). DAPI were used to stain the nucleus. Fluorescence intensity over area measurements were performed with ImageJ 1.53e (https://imagej.nih.gov/ij/).

### Western Blot Analysis

Protein samples were separated on SDS-PAGE on 4–15% gels and transferred to polyvinylidene difluoride (PVDF; BioRad) membranes. Membranes were blocked for 1 hr at room temperature in 5% milk or BSA in TBS-Tween-20 (pH 7.6) and then incubated with primary antibodies overnight at 4°C. Subsequently, the membranes were incubated with secondary antibodies [horseradish peroxidase-conjugated anti-rabbit immunoglobulin G or anti-mouse IgG; 1:1,000; CST] at 37°C for 1 hr. Signals were detected and quantified using Odyssey® Fc Imaging System (LI-COR Biosciences, model number 2800). Detailed information for each antibody is provided in the (Supplementary Table 2).

### Exosomal miRNA Reverse Transcription for miRNA Profiling

Exosomal miRNA was isolated using miRNeasy Mini Kit (Qiagen) and reverse transcribed using miScript II RT kit according to the manufacturer's protocol. miRNA PCR array was performed using the miScript SYBR Green PCR Kit on custom-printed 96-well miScript miRNA PCR fibrosis array (Qiagen, Catalog #MIRN-117Z) as per the manufacturer's instructions. The miScript miRNA real-time PCR was performed using QuantStudio3 (Applied Biosystem). Ct values were normalized with housekeeping genes (SNORD61, SNORD68, SNORD95, SNORD96A, and RNU6B) provided in the custom plate.

### TaqMan miRNA Assay for miRNA Array Validation

Significantly altered miRNAs were further confirmed by TaqMan miRNA assays (Applied Biosystems). The expression of each miRNA was normalized using miR-423-5p (rno481159_mir) as per the manufacturer's instructions (http://tools.thermofisher.com/content/sfs/manuals/100027897_TaqManAdv_miRNA_Assays_UG.pdf). Quantitative PCR data were expressed as “fold-change” relative to control levels.

### Bioinformatic Analysis

Computational predictions of target genes were performed using the miRNet-an integrated platform linking miRNAs, targets and functions (https://www.mirnet.ca/miRNet/home.xhtml), a prediction tool available online.

### miR-Modulation and Exosomes Isolation

NRVFs were seeded into six-well-plates and grown to 60–70% confluence and transfected with Rno-miR-200a-3p inhibitor or control (scramble miRNA) (all from ThermoFisher) using Lipofectamine-2000 Transfection Reagent (ThermoFisher, Catalog #11668030) in Opti-MEM Reduced Serum Medium (ThermoFisher, Catalog #31985070). After 8 h, media were changed to DMEM 4.5 g/L (without antibiotic/antimycotic) and cells were treated with BSA or TGF-β1. Finally, media were collected for exosome isolation and cells were harvested for biochemical analysis. The exosomes isolated in this step were used to treat endothelial cells to study its effect on endothelial cell biology and function.

### Angiogenesis Array

Angiogenesis-related proteins were analyzed using the Proteome Profiler™ Mouse Angiogenesis Antibody Array (R&D Systems), following the manufacturer's protocol. We collected proteins from endothelial cells treated with PBS or with exosomes collected from NRVFs treated with either BSA or TGF-β1 (under the same conditions as functional and other experiments). Signals were detected and quantified using Odyssey® Fc Imaging System.

### Statistical Analyses

Statistical analyses were carried out using Prism software (Graph Pad). Unpaired, 2-tailed Student's *t*-test was used for determining significance between 2 groups, while One-way ANOVA followed by Turkey *post-hoc* test was applied to calculate significance when more than 2 groups were involved. *P* < 0.05 were considered significant. Data are presented as mean ± s.e.m. of 4 to 6 biological replicates unless otherwise mentioned.

## Results

### TGF-β1 Treatment Activates Neonatal Rat and Adult Mouse Cardiac Fibroblasts

**Figure 1:**
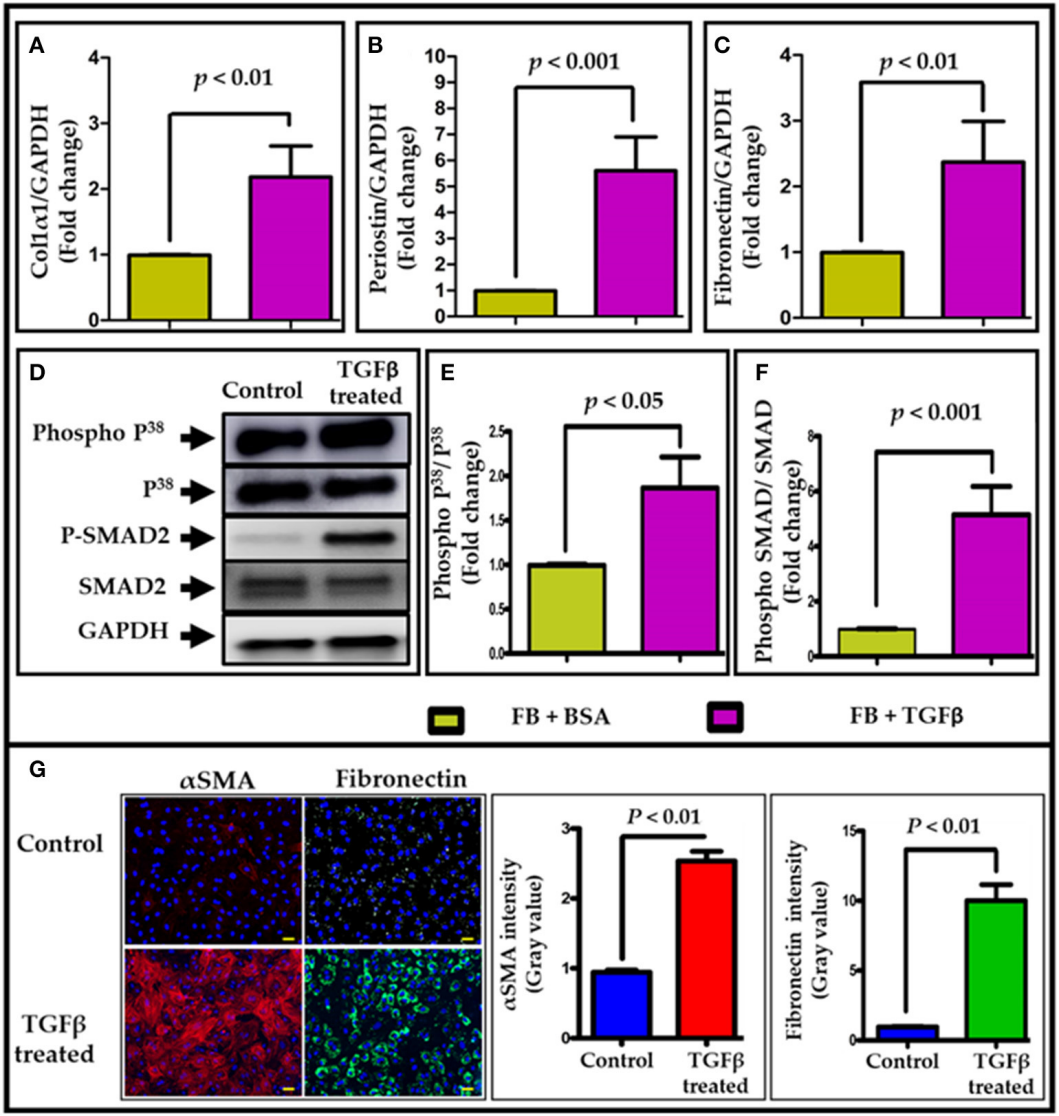
TGFβ treatment activates cardiac fibroblasts. TGFβ treatment significantly upregulated pro-fibrotic genes expression, including **(A)** collagen1α (Col1α), **(B)** periostin (Postn), and **(C)** fibronectin (Fn1) in NRVF cells. In addition, an increased phosphorylation of p38 and SMAD2 **(D–F)** was observed in TGFβ-activated NRVFs. GAPDH was used as loading control for both mRNA and protein expression analysis. **(G)** Immunostaining of BSA and TGFβ-treated fibroblasts with αSMA (red)/fibronectin (green)/DAPI (blue). Fibrotic markers were significantly upregulated upon TGFβ treatment. Images **(G)** were taken at 400X and scale bar is 100 μm. *P* < 0.05 was considered as statistical significance.

TGF-β1 (hereafter TGFβ) is known to enhance fibroblast activation and to mediate cardiac fibrosis; thus, we measured fibrotic markers in both BSA- and TGFβ treated NRVF cells. Real time qRT-PCR data revealed that *collagen type 1*, α*1* (Figure 1A), *periostin* (Figure 1B) and *fibronectin* (Figure 1C) mRNA expression was significantly increased in TGFβ-treated NRVFs compared to BSA treatment. Furthermore, immunocytochemistry analysis was performed to determine whether TGFβ activates fibroblasts to myofibroblasts. Immunostaining data suggested that TGFβ significantly induced trans-differentiation of fibroblast to myofibroblast as shown by increased expression of αSMA (Red) and fibronectin (Green) (Figure 1G). Increased phosphorylation of proteins p38 and SMAD2/3 (Figures 1D–F) further indicated the activation of TGFβ signaling pathway in TGFβ-treated fibroblasts as compared to BSA-treated cells. To validate the effects of TGFβ on adult mouse fibroblasts, we isolated and cultured adult mouse cardiac FBs and treated with TGFβ. Both qPCR and western data revealed similar results as with NRVFs (Supplementary Figure 1). These data together suggest that TGFβ treatment activates cardiac fibroblasts and that both adult mouse and neonatal rat FBs respond equally well to TGFβ treatment. In further experiments, we used neonatal rat fibroblasts because this system allows us for efficient collection of large quantities of exosomes.

### Characterization of NRVF-Derived Exosomes

**Figure 2:**
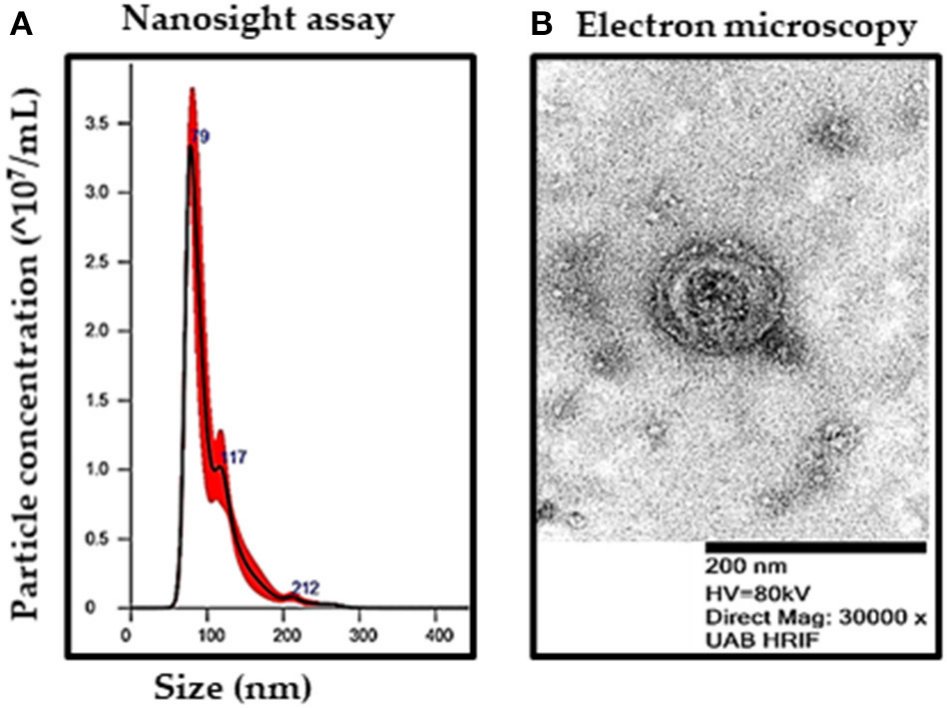
Characterization of exosomes from fibroblast-conditioned media after TGFβ treatments. **(A)** Size and number of exosomes were determined by nanoparticle tracking analysis. **(B)** Representative image by transmission electron microscopy (magnification 30,000X) revealed the presence of double-membrane bound, cup-shaped exosomes.

We isolated exosomes from the conditioned media derived from control and TGFβ-treated NRVFs (TGFβ-FB-Exo) following ultracentrifugation. Nanoparticle tracking analysis and electron microscopic data validate the quality of isolated exosomes, as shown by double membrane cup-shaped structure with average size of 90 nm (Figures 2A,B). Notably, TGFβ treatment significantly enhanced the number of exosomes secreted from myofibroblats (Supplementary Figure 2).

### Myofibroblasts-Derived Exosomes Impair Endothelial Function

**Figure 3:**
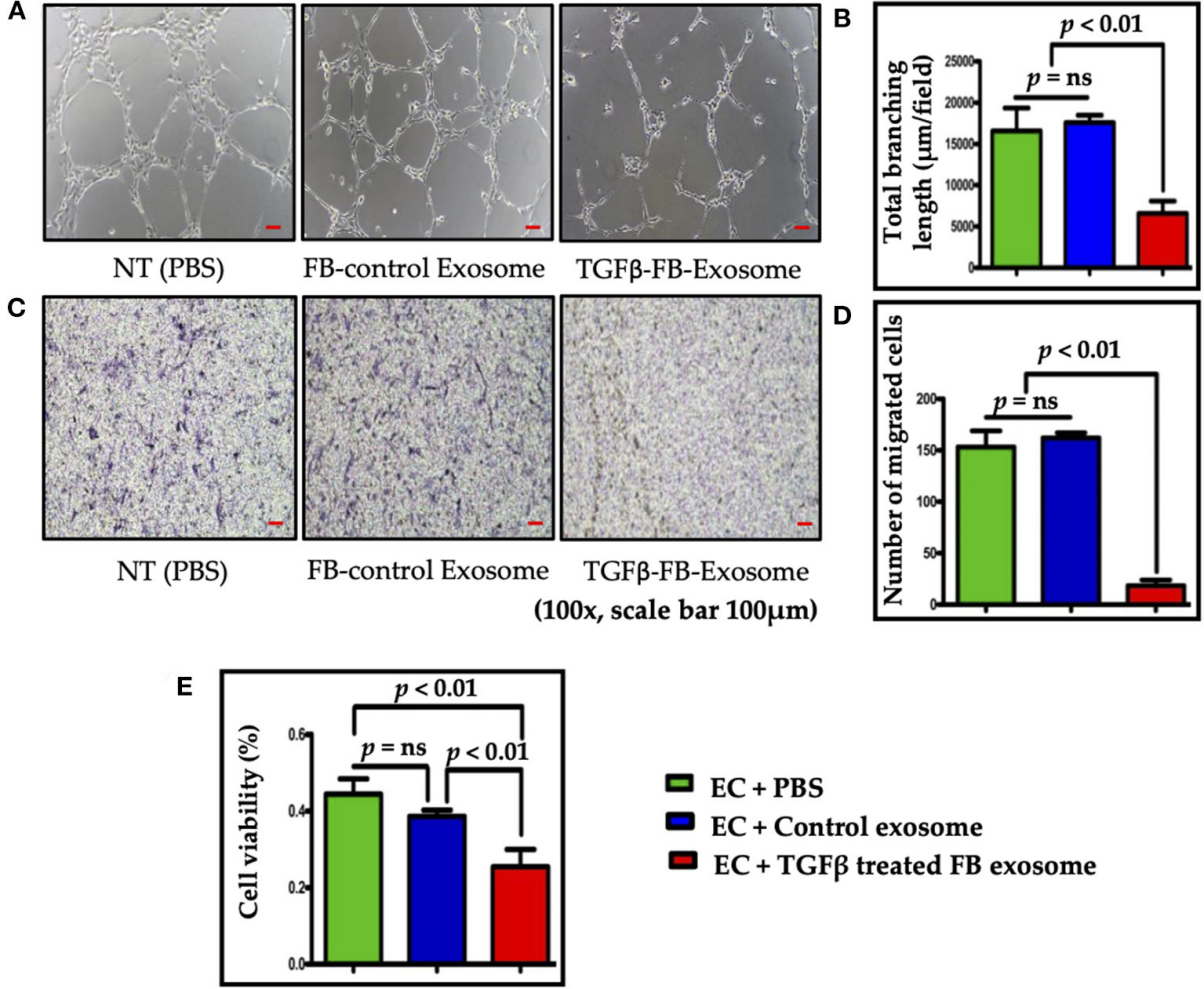
Exosomes derived from TGFβ-activated fibroblast reduced endothelial cell function and viability. **(A,B)** Tube formation assay for endothelial cell angiogenesis function; representative images are shown. For data analysis the length of branch points was measured using NIH ImageJ software, and the mean length is represented in the graph. Activated FB-derived exosome treatment significantly impaired tubulogenic potential of heart ECs. **(C,D)** Images of ECs with Giemsa stain for evaluating cell migration in a Boyden chamber. As anticipated, activated FB-derived exosomes significantly reduced EC migration as compared to control. **(E)** MTT assay to measure endothelial cell viability. Endothelial cell viability (%) was significantly reduced after treatment with activated FB-derived exosomes. *P* < 0.05 was considered as statistical significance.

To determine the direct role of activated NRVF-exosomes on ECs, mouse primary cardiac endothelial cells were treated with PBS or exosomes isolated from NRVFs (which had been treated with either BSA or TGFβ). *In vitro*, the Matrigel tube formation assay was performed to evaluate the effect of exosomes on angiogenic potential of mouse ECs. Exosomes derived from TGFβ treated myofibroblasts notably impaired endothelial cell angiogenic potential as compared to control exosomes (Figures 3A,B). ECs have immense potential to migrate toward a nutrient gradient. To measure the effect of TGFβ-exosomes on EC migration, ECs were cultured in Boyden chamber after above treatments. Intriguingly, EC migration was drastically reduced by TGFβ-FB-exosome treatment as compared to control exosome treatment (Figures 3C,D). Further, the effect of myofibroblast-derived exosomes (TGFβ-FB-Exo) on EC viability was measured using MTT assay Kit. MTT assay data revealed that control exosome treatment only slightly altered cell viability; however, a significant decrease in EC viability was observed in TGFβ-FB-exosome-treated cells (Figure 3E). Together, these data suggest that activated fibroblast-derived exosomes significantly hinder endothelial cell functions.

### Myofibroblast-Derived Exosomes Induce Endothelial Cell Death

**Figure 4:**
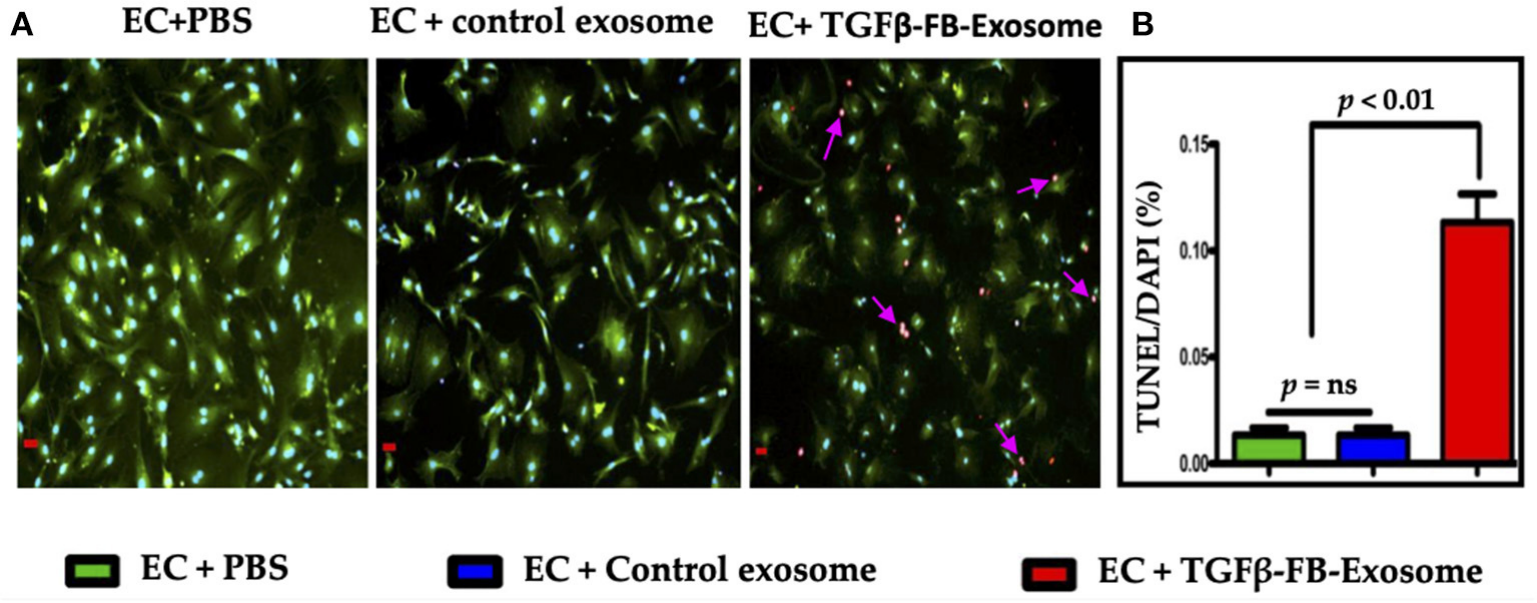
Myofibroblasts-derived exosomes induced endothelial cell death **(A,B)**. Primary cardiac endothelial cells were isolated from the C57BL/6 mice and were treated with exosomes derived from TGFβ-treated fibroblasts for 48 h. TUNEL staining was performed in 4% PFA fixed cells as per manufacturer's instruction. ECs were counterstained with CD31/PECAM (green; EC surface marker) antibody. Images **(A)** were taken at 400X and scale bar is 100 μm. *P* < 0.05 vs. control exosome and PBS treatment.

Our data suggest that myofibroblast-derived exosomes significantly reduced EC viability. Therefore, next we checked the effect of activated FB-derived exosomes on endothelial cell death via terminal deoxynucleotidyl transferase nick-end labeling (TUNEL) assay. ECs treated with activated fibroblast-derived exosomes exhibited significantly increased numbers of TUNEL-positive cells as compared to the control exosome-treated group (Figure 4). Taken together, our data clearly advocate that activated cardiac fibroblast-derived exosomes contain some critical factors which impair endothelial cell function.

### Myofibroblast-Derived Exosomes Impair Angiogenesis-Associated Genes/Proteins Expression

**Figure 5:**
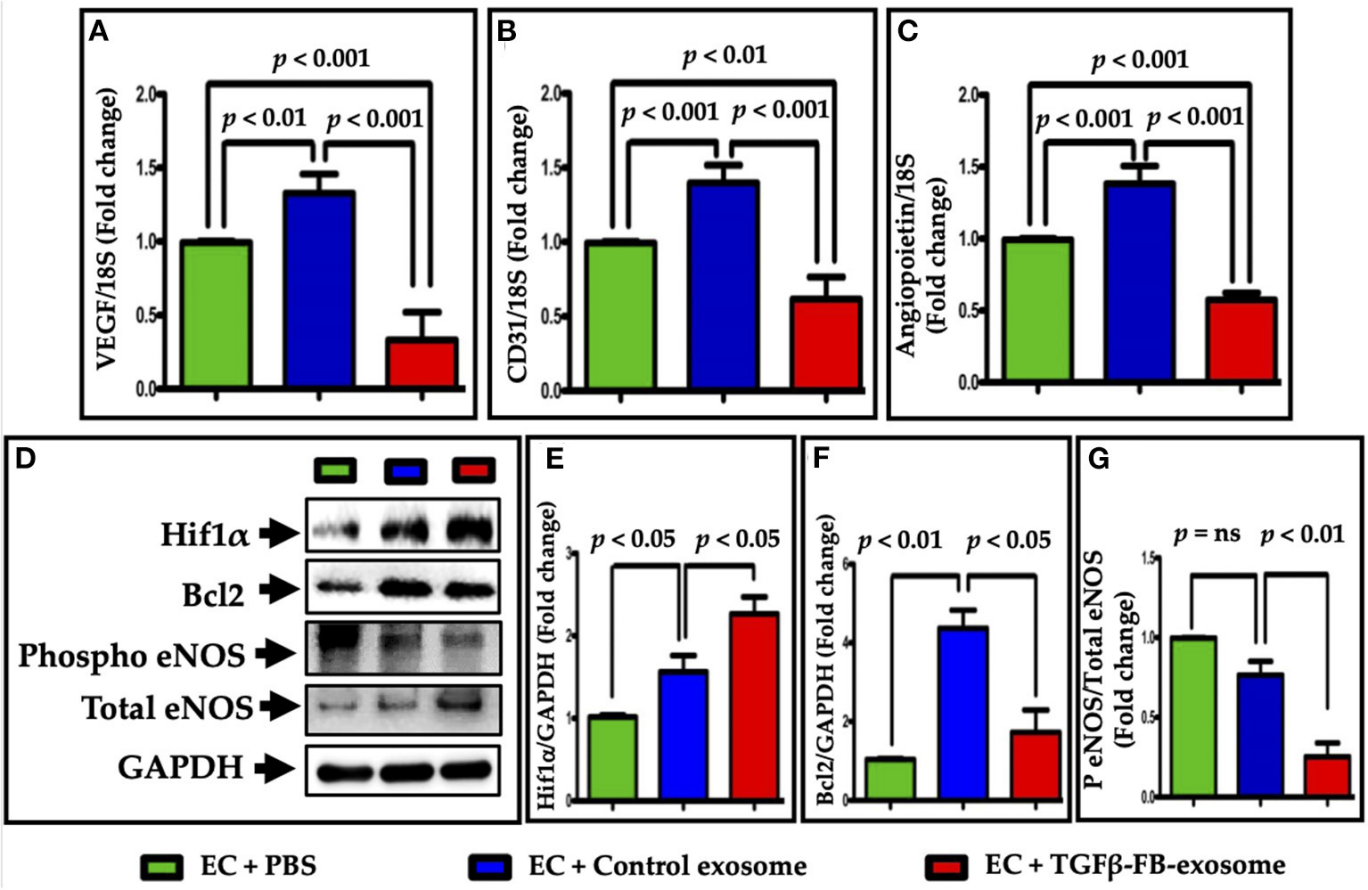
Myofibroblast-derived exosomes impaired EC function-related genes and proteins expression. The mRNA expression of **(A)** VEGF-A, **(B)** CD31, and **(C)** angiopoietin1 were downregulated in the primary endothelial cells after treatment with TGFβ-activated exosomes, as analyzed by real time qPCR. **(D–G)** Protein levels of HIF1α, eNOS, and Bcl2 were measured. Expression of eNOS and Hif1α were significantly altered by TGFβ-FB-exosomes as compared to control exosomes, but in opposite manner. Bcl2 expression was also decreased in TGFβ-FB-exosomes treated endothelial cells. *P* < 0.05 was considered as statistical significance.

To determine the effect of activated fibroblast-derived exosomes on endothelial cell biology, we assessed expression of important endothelial cell marker genes following the same series of treatments as used for the EC function assays. The RT qPCR data showed that vascular endothelial growth factor-A (VEGF-A) (Figure 5A), CD31 (Figure 5B), and angiopoietin1 (Figure 5C) gene expression was significantly reduced in ECs treated with TGFβ-activated FB-derived exosomes (TGFβ-FB-Exo) compared to control exosomes. The control exosomal treatment to the ECs upregulated VEGF-A, CD31, and angiopoietin1 (APO1) mRNA expression slightly as compared to PBS group. It is possible that control exosomes secreted by undifferentiated fibroblasts carry some factors which support endothelial cell growth. Furthermore, western blot analysis revealed that eNOS (an important endothelial functional protein) expression was higher in control exosome-treated ECs compared to TGFβ-activated exosomes (Figures 5D,G). Hypoxia inducible factors-1α (HIF1α) is known for its role in fibrosis and in endothelial dysfunction (27, 28). Therefore, we examined Hif1α protein expression and found a significant increase in ECs treated with TGFβ-activated exosomes compared to PBS (Figures 5D,E). This is in line with the previous study that hypoxia induces HIF1α, arginase type II (Arg II), and ICAM-mediated signaling in ECs which ultimately leads to endothelial dysfunction (28). The Bcl2 protein level was increased by control exosomes compared to PBS, while TGFβ-activated exosome had significantly decreased its expression (Figures 5D,F). These data suggest that angiogenesis and cell migration of ECs are impaired by TGFβ-activated exosomes.

### TGFβ Treatment Alters Fibrosis-Associated miRNAs in Myofibroblast-Derived Exosomes

**Figure 6:**
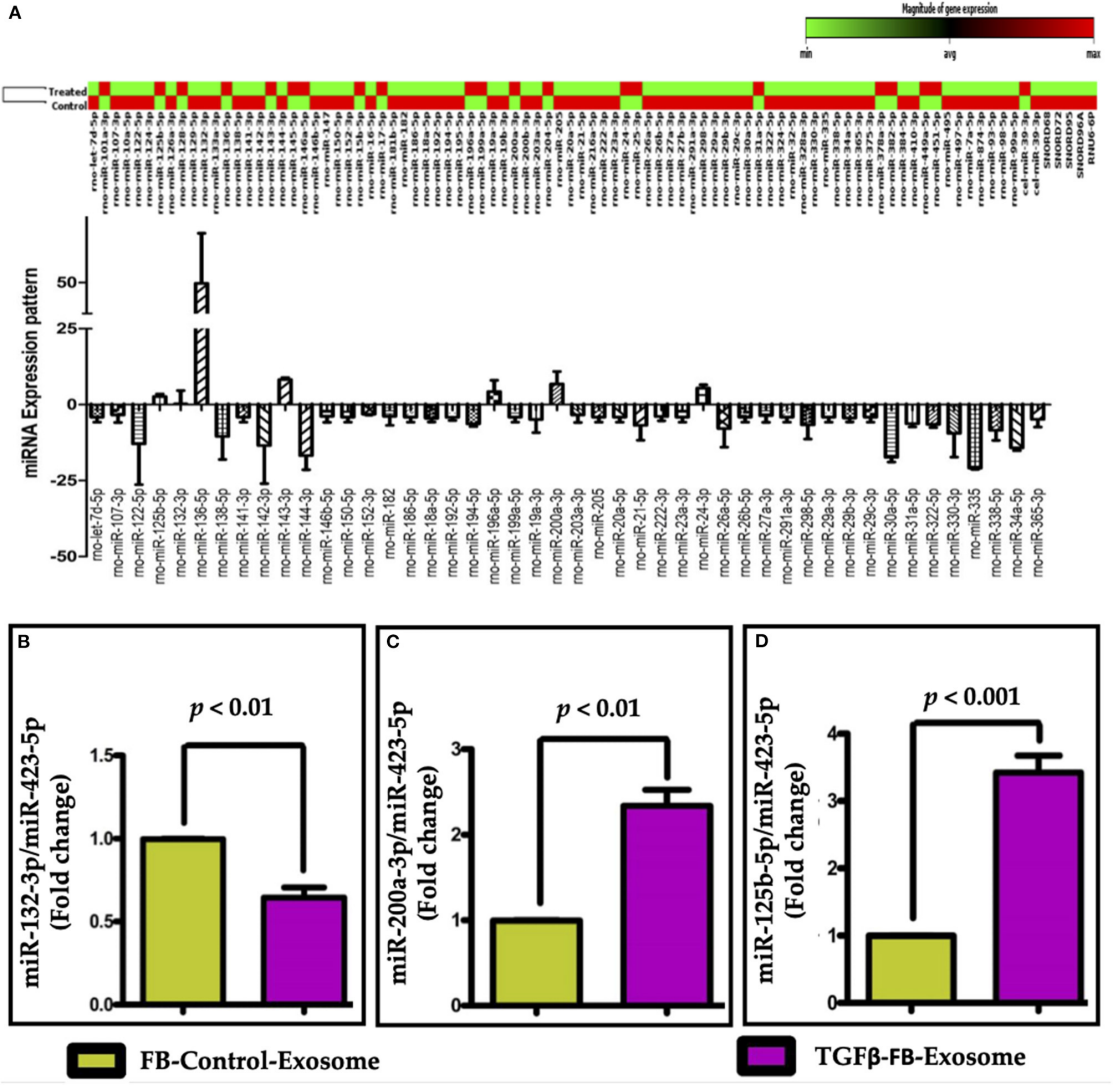
Fibrosis miRNA array along with expression profile and validation of selected miRNAs in exosomes. **(A)** Fibrosis-miRNA array was performed in neonatal rat fibroblast-derived exosomes after TGFβ activation. Heat map of fibrotic miRNA array data suggest an altered expression of miRNAs in TGFβ-activated FB-derived exosomes as compared to control exosome. The change in expression of selected miRNAs was further confirmed by qPCR by comparing the expression of miRNAs **(B)** miR-132-3p, **(C)** miR-200a-3p, and **(D)** miR-125b-5p, in activated-FB-derived exosomes. *P* < 0.05 vs. control exosome and PBS treatment.

To understand the molecular mechanisms that lead to exosome-mediated endothelial cell dysfunction, next we explored the contents and factors packaged in both control and activated fibroblast-derived exosomes. The role of microRNAs has been substantially reported in both profibrotic and endothelial dysfunction processes. Therefore, we performed the fibrosis-associated miRNA array assay in the exosomes-derived from control and TGFβ-treated cells. We analyzed miRNA from control and TGFβ-treated exosomes in the miScript miRNA PCR Array Rat Fibrosis Pathway focused panels (Qiagen) and found that expression levels of many fibrosis-related miRNAs were altered in TGFβ-treated FB derived exosomes as compared to control exosomes (Figure 6A). Using pathway-based analysis miRNet—an integrated platform linking miRNAs, targets, and functions, we screened and selected miR-132-3p, miR-200a-3p, and miR-125b-5p miRNAs which may play important roles in regulation of endothelial cell function. We also performed a miScript miRNA Mouse Fibrosis Array on exosomes isolated from adult mouse cardiac fibroblasts. We found a similar trend in exosomes from mice after TGFβ treatment of FB (Supplementary Figure 3). To validate our array data, we performed RT-qPCR using selective primers for miR-132-3p, miR-200a-3p, and miR-125b-5p to measure their expression in control- and TGFβ-treated exosomes. TGFβ-treated FB-derived exosomes contained significantly reduced levels of miR132-3p as compared to control exosomes. Interestingly, TGFβ treatment significantly increased the packaging of miR-200a-3p and miR-125b-5p in myofibroblasts when compared to the control exosomes (Figures 6B–D). We further analyzed exosomes for TGFβ mRNA content and did not find TGFβ in exosomes. These data clearly suggest that an altered packaging of miRNAs occurs in activated fibroblast-derived exosomes as compared to control exosomes.

### Myofibroblasts-Derived Exosome-Induced Endothelial Cell Dysfunction Is Mediated by miR-200a-3p

**Figure 7:**
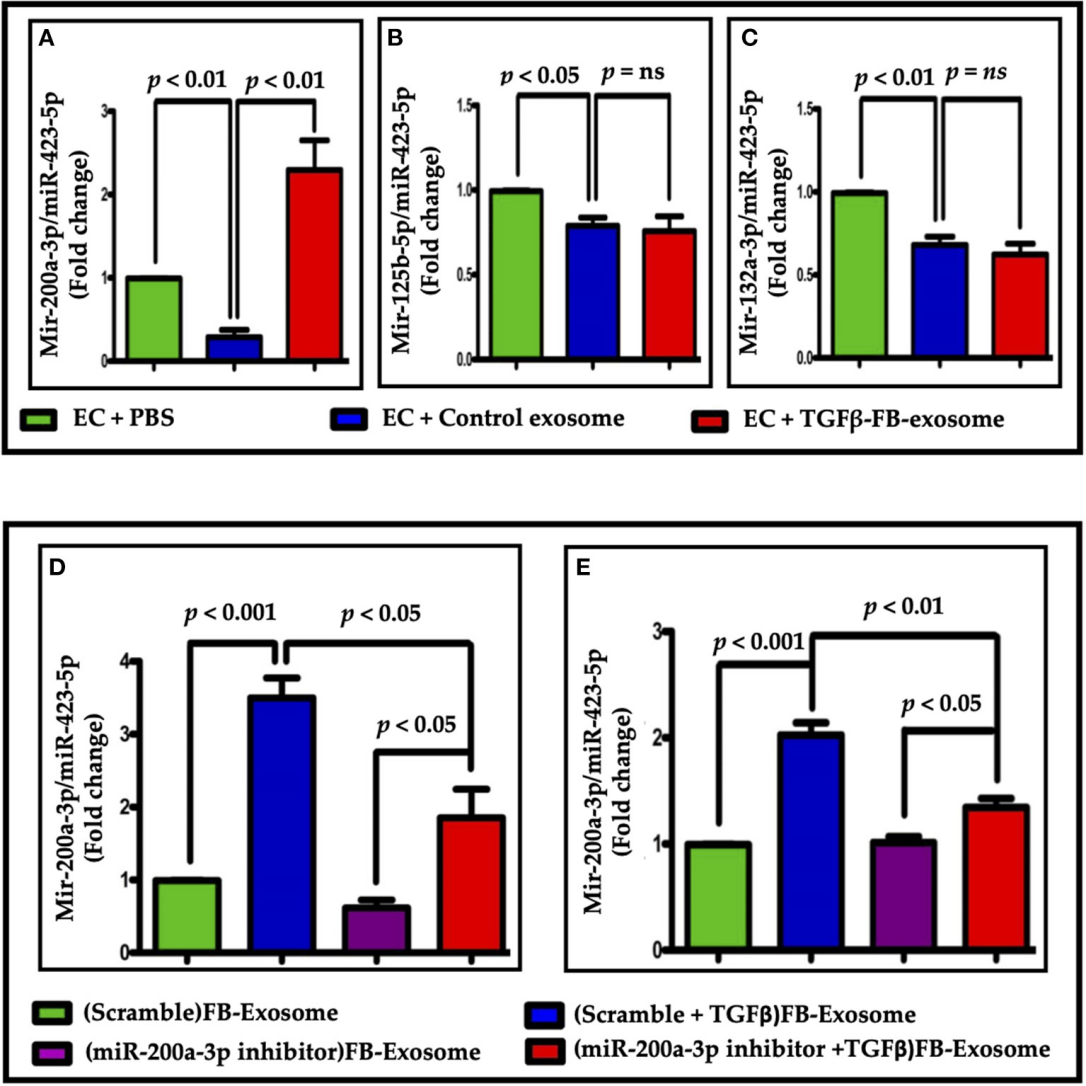
Altered miRNA (miRNA-200a-3p) packaging was seen in myofibroblast-derived exosomes. **(A–C)** Altered miRNA expression was found in ECs after treatments. Only miR-200a-3p expression showed a significant difference when treated with TGFβ-FB-exosomes. **(D,E)** Analysis of miR-200a-3p expression in exosomes **(D)** or EC **(E)** following miR200a-3p inhibition in parent cells. **(E)** miR-200a-3p expression was significantly reduced in endothelial cells treated with modified exosomes in comparison to Exo-TGFβ treatment alone. *P* < 0.05 vs. control exosome and PBS treatment. “ns”, not statistically significant.

Previous studies have suggested the role of miR-200a-3p in endothelial to mesenchymal transition (29). Our data suggests that the packaging of miR-200a-3p was significantly increased in TGFβ-treated exosomes when compared to control (Figures 7A–C). To determine whether the impaired EC function is mediated by miR-200a-3p packaged in myofibroblast-derived exosomes, we inhibited miR-200a-3p expression in TGFβ treated FBs using an antagomir for miR-200a-3p. In support of our hypothesis, antimiR-200a-3p transfection significantly reduced the packaging of miR-200a-3p in exosomes-derived from TGFβ treated FBs as compared to the corresponding scramble group (Figure 7D). Treatment of ECs with exosomes from the altered FBs reflected a similar reduction in miR-200a-3p content in corresponding ECs (Figure 7E). Our data suggest that by altering fibroblast content using molecular tools, we can alter the parent cell-derived exosomal composition.

### Myofibroblast-Derived Exosomes Worsen Endothelial Cell Dysfunction by Delivering miR-200a-3p

**Figure 8:**
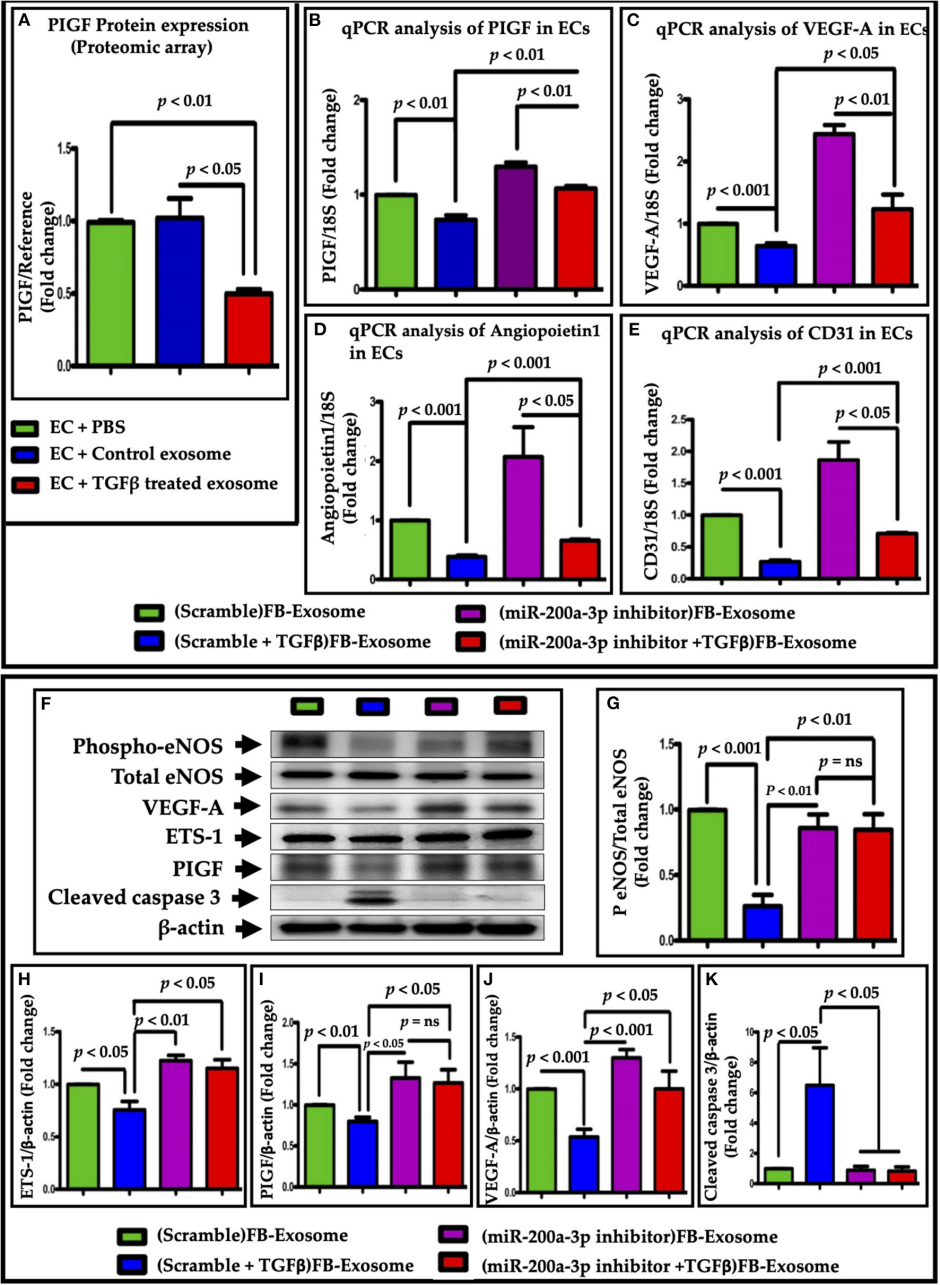
miR-200a-3p packaged in fibroblast-derived exosomes altered expression of endothelial functional genes via PIGF growth factor inhibition. **(A)** ELISA-based angiogenesis proteomic profiling revealed a greatly reduced expression of PIGF. **(B)** PIGF expression was increased in ECs upon treatment with exosomes with inhibited miR-200a-3p. qPCR data showing an improved endothelial function, as assessed by **(C)** VEGF-A, **(D)** angiopoietin1 and **(E)** CD31 genes expression, upon treatment with miR-200a-3p inhibited exosomes. **(F–K)** Western blot analysis was performed in the cell lysate isolated from ECs treated with modified exosomes (inhibited miR-200a-3p). Cleaved caspase 3 expression **(F,K)** showed that endothelial cell death was also observed in FB- TGFβ-Exo treated cells which was significantly recovered after miR-200a-3p inhibition. *P* < 0.05 vs. control exosome and PBS treatment. “ns”, not statistically significant.

To understand the molecular mechanism for miR-200a-3p mediated impaired EC function, we performed an ELISA-based angiogenesis array in ECs after exosome treatments. In addition of many other altered factors, the expression of placental growth factor (PIGF), which is very important for endothelial biology and function (30), was greatly reduced in ECs after TGFβ-treated FB-derived exosome treatment (Figure 8A, Supplementary Figure 4). To investigate whether miR-200a-3p mediates endothelial dysfunction via PIGF, we measured PIGF expression in ECs after treatment with exosomes-derived from scrambled control- or miR-200a-3p antagomir-treated NRVFs with or without TGFβ. Interestingly, PIGF expression was significantly restored after miR-200a-3p inhibition (Figure 8B). Further, we assessed the expression of EC functional markers genes using RT qPCR. As expected, endothelial cells treated with activated fibroblast-derived exosomes showed significantly reduced VEGF-A, Angiopoietin1 and CD31 expression. These data were further corroborated by miR-200a-3p inhibition in TGFβ-activated fibroblasts. miR-200-3p inhibition noticeably improved VEGF-A, Angiopoietin1 and CD31 expression as compared to activated FB exosomes alone in ECs (Figures 8C–E). These data suggest the direct involvement of FB-derived miR-200a-3p on endothelial cell function via PIGF.

To confirm the effect of miR-200a-3p at the protein level of eNOS, VEGF-A, ETS and PIGF, we performed western blot analysis of these proteins in endothelial cell lysates following treatment with FB-derived exosomes. Interestingly, exosomes derived from miR-200a-3p antimiR treated FB significantly restored the eNOS and VEGF-A expression as compared to TGFβ treated cells (Figures 8F,G,J). These data further support the direct involvement of FB-derived miR-200a-3p on endothelial cell function. Since ETS-1 is the well-known regulator of expression of several downstream targets in ECs that promote an angiogenesis, including the VEGF-A receptors through PIGF induction (31), next we evaluated its expression. As expected, modified exosomes (derived from FB treated with antimiR-200a-3p and TGFβ) significantly restored ETS-1 and PIGF protein expression as compare to the cells which were treated with exosomes derived from TGFβ treated FB (Figures 8F–I). The reduction of PIGF by miR-200a-3q inhibitor was less than lowering of VEGF-A. This may be because the regulation of one protein at genetic level is determined by many factors. The degree of alteration depends on the translational fidelity of each gene which heavily depends on the demands of the cell. Interestingly, Bcl2 levels was not significantly altered with TGFβ-treated exosomes (Figures 5D,F). At molecular level, multiple signaling pathways involved in cell death process. To determine whether FB-TGFβ-Exosome mediated cell death is independent of Bcl2, we checked cleaved caspase 3 level in FB-TGFβ-Exo treated endothelial cells. Our data suggests that FB-TGFβ-Exo treatment-induced endothelial cell death (Figures 8F,K) is mediated by caspase 3 signaling pathways and miR-200 inhibition using antimir-200 attenuate it. This is in accordance with the Hoffmann et al. who showed increased caspase 3 activity and significantly enhanced apoptotic induction in eNOS knockout endothelial cells (32).

## Discussion

Cardiac fibroblasts play an important role in regulating heart physiology during injury (33). Emerging evidence suggests that exosomes released from cardiac fibroblasts are one of the major components contributing to cardiomyocyte hypertrophy (22). Importantly, fibroblasts play an important role in angiogenesis. Under normal physiologic conditions, fibroblasts secrete soluble growth factors such as VEGF-A, transforming growth factor-β (TGFβ), placental growth factor (PIGF), and others to promote healthy cardiac function (34, 35). In the recent past, several published papers have demonstrated direct involvement of fibroblasts in EC-mediated vessel formation (36–38). Thus, any alteration in cardiac fibroblast behavior may modulate endothelial biology and function. In the present study, we have shown that activated cardiac fibroblasts (myofibroblasts) secrete altered exosomes. We found that miR-200a-3q secreted from TGFβ-activated fibroblasts alters angiogenic potential, proliferation, and migration of endothelial cells. Furthermore, reduced viability and increased apoptosis were observed in endothelial cells treated with myofibroblast-derived exosomes. Finally, we found that excessive packaging of miR-200a-3p in myoFB-derived exosomes modulates PIGF-dependent VEGF-A signaling and thus induces endothelial cell dysfunction. In accordance, inhibition of miR-200a-3p in FBs and in FB-derived exosomes significantly restored endothelial function.

Cardiac fibroblasts account for over 60% of cardiac cells (39). Under normal condition, FB produce extracellular matrix proteins and many growth factors that support the functions of many neighboring cells. However, during disease states, fibroblasts become activated and worsen adverse cardiac remodeling. Recent studies have suggested that fibroblasts play a significant role in cardiac remodeling and heart failure (33). Growing evidence suggests that different heart cell types (cardiac myocytes, fibroblasts, endothelial cell, and immune cells) interact with each other via extracellular vesicles called exosomes. Deep analysis of exosomes suggests that these tiny vesicles carry many important signaling molecules and regulate the recipient cell biology and function. Recently, Bang et al. showed that miR-21 carried by cardiac fibroblast-derived exosomes significantly impaired cardiac myocyte function in pressure-overloaded myocardium (22). In our study, we found that activated FB-derived exosomes impaired endothelial cell function by reducing angiogenic and migration potential as well as promoting endothelial cell death. Consistent with other studies, our pathway-based miRNA array analysis data suggest an altered packaging of miRNAs in activated fibroblast-derived exosomes. The most common altered miRNAs in both adult and neonatal cells derived exosomes were miR-132-3p, miR-200a-3p, and miR-125b-5p.

**Figure 9:**
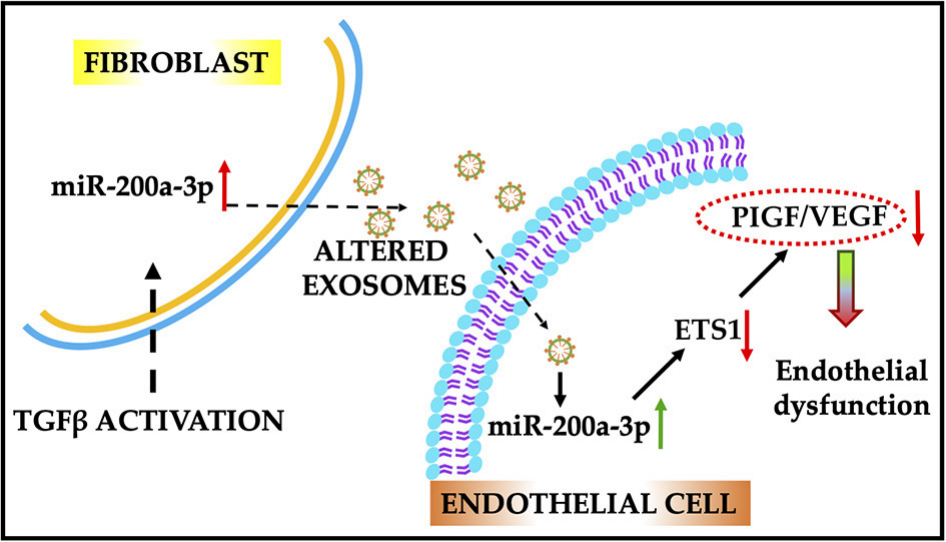
Schematic illustration of the proposed role of miR-200a-3p/ETS1/VEGF-A signaling cascade in endothelial dysfunction. Activated fibroblast-derived exosomes enriched with miR-200a-3p induce endothelial dysfunction by altering the VEGF-A/PIGF signaling. Alteration in miR-200a-3p expression using antimir therapy improved endothelial cell function. ETS1, E26 transformation-specific sequence factor-1; VEGF-A, vascular endothelial growth factor-A; PIGF, placental growth factor.

The role of the miRNA 200 family in endothelial to mesenchymal transition process as well as on endothelial function is well-established (40, 41). Zhang et al. has suggested an increased expression of miR-200c in arteries from diabetic mice and from patients with diabetes (41). Remarkably, downregulation of miR-200b increases VEGF-A expression, promotes angiogenesis and ameliorates diabetic retinopathy (42). Here, we found that activated fibroblasts can produce more miR-200a-3p and shuttle it to neighboring cells via exosomes. This notion is further supported by the data where we showed the abundance of miR-200a-3p in endothelial cells treated with activated fibroblast-derived exosomes. Intriguingly, our data have evidently shown that treatment of ECs with modified exosomes significantly rescued the functional property of endothelial cells. Consistent with our results, several other studies have shown a role of the miR-200 family in endothelial cell dysfunction. miR-200 family miRNAs regulate vascular endothelial growth factor expression and angiogenesis (42). Elevated miR-200b induced inflammation in vascular smooth muscle cells (43). In addition, miR-200c plays a critical role in diabetes-associated endothelial dysfunction (41). Inhibition of miR-200c also restores endothelial function in diabetic mice through upregulation of ZEB1 (44). Many reports have shown that micro RNA from miR-200 affects endothelial cell function by suppressing ZEB1 (41, 44) and subsequent upregulation of COX2 (45, 46). The miR-200 family also inhibits cell migration (47). Our data support this fact that miR-200 significantly inhibits migration of ECs. Apart from the miR-200/ZEB1/COX2 signaling axis, miR-200b negatively regulates VEGF-A and its receptors Flt-1 and KDR and thus interferes with angiogenesis (48). Other studies confirmed that miR-200b directly targets the angiogenic response via silencing of Ets-1 which regulates the expression of several downstream targets in ECs that promote an angiogenic phenotype, including the VEGF-A receptors (49). Our angiogenesis protein array data showed that expression of placental growth factor (PIGF) was greatly reduced when we transfected TGFβ-FB-Exo to endothelial cells. PIGF is a member of the VEGF-A family of growth factors and induces monocyte activation, migration, and production of inflammatory cytokines and VEGF-A (50). Autiero et al. suggested that endothelial cells can enhance their own responsiveness to VEGF-A by releasing PlGF (31). Basically, PIGF induces VEGF-A-driven angiogenesis. Ets-1 is negatively regulated by miR-200 family (b) in human microvasculature endothelial cells (HMEC)s and is a proven target of miR-200b (49). In accordance with our finding, previous studies suggest that overexpression of miR-200b (using miR-200b mimic) enhanced angiogenesis in endothelial cells (49). They also observed that ETS-1 is a key mediator of cell migration and Matrigel tube formation. Furthermore, the promoter regions of the VEGF-A receptor, a master regulator of endothelial gene transcription, contains Ets-1 binding sites, and VEGF-A itself may enhance transcription of other angiogenic growth factors by acetylation of Ets-1 (51, 52). PlGF is a member of the VEGF superfamily secreted by cardiomyocytes and cardiac microvascular endothelial cells. Impaired angiogenesis was reported in PlGF knockout mice after pressure overload (53). PIGF also upregulates expression of VEGF-A by binding VEGF-R1 (54). Based on these publications, our findings also suggest that miR-200a-3p could regulate endothelial cell function via the Ets-1/PIGF/VEGF-A signaling pathway. In conclusion, we report that activation of fibroblasts promotes endothelial dysfunction via an increase in the levels of miR-200a-3p in exosomes (refer to Figure 9 for proposed mechanism of action). The understanding of the miR-200a-3p/Ets-1/PIGF/VEGF-A axis provides new insights in the endothelial dysfunction mechanisms involved during cardiac fibrosis.

## Data Availability Statement

The raw data supporting the conclusions of this article will be made available by the authors, without undue reservation.

## Ethics Statement

The animal study was reviewed and approved by Institutional Animal Care and Use Committee, The University of Alabama Birmingham.

## Author Contributions

PR and SV designed the study. PR, HP, ZC, and SV discussed the study. PR performed the experiment, analyzed data and wrote manuscript. SV edited the manuscript. All authors reviewed and edited the manuscript.

## Conflict of Interest

The authors declare that the research was conducted in the absence of any commercial or financial relationships that could be construed as a potential conflict of interest.
