# Supplementary material for: Myofibroblast-Derived Exosome Induce Cardiac Endothelial Cell Dysfunction
Source: Front Cardiovasc Med. 2021 Apr 23;8:676267. doi: 10.3389/fcvm.2021.676267 (PMC8102743; doi:10.3389/fcvm.2021.676267)
Supplement: Supplementary file 1 [file Table_1.DOCX]

*List of Primers used*

| **S. No.** | **Gene** | **Primer’s Assay ID (Rat)** |
| --- | --- | --- |
| 1. | Col1a1 | Rn01463874_g1 |
| 2. | αSMA (or Acta2) | Rn01759928_g1 |
| 3. | GAPDH | Rn01775763_g1 |
| 4. | Periostin (or Postn) | Rn01494627_m1 |
| 5. | Fibronectin | Rn00569575_m1 |
| 6. | VEGF-A | Rn01511602_m1 |
| 7. | 18S | Rn03928990_g1 |
| **S. No.** | **Gene** | **Primer’s Assay ID (Mice)** |
| 8. | Col1a1 | Mm00801666_g1 |
| 9. | αSMA (or Acta2) | Mm00725412_s1 |
| 10. | GAPDH | Mm9999915_g1 |
| 11. | Periostin (or Postn) | Mm01284919_m1 |
| 12. | Fibronectin | Mm01256744_m1 |
| 13. | VEGF-A | Mm00437306_m1 |
| 14. | 18S | Mm03928990_g1 |
| 15. | CD31 (or Pecam1) | Mm01242576_m1 |
| 16. | Angiopoietin | Mm00456503_m1 |
| 17. | eNOS (Nos3) | Mm00435217_m1 |
| 18. | Hif1α | Mm00468869_m1 |
| 19. | TGFβ | Mm01178820-m1 |
| 20. | miR-132-3p | 480919_mir |
| 21. | miR-200a-3p | 478490_mir |
| 22. | miR-125b-5p | 480907_mir |
| 23. | miR-423-5p | 478090_mir |
| 24. | PIGF | Mm00435613_m1 |

## Supplementary Table S1. *We have enlisted here the assay ID of all the primers used in this work. All the primers were purchased from the “Thermo Fisher Scientiﬁc”.*
