# Supplementary material for: Myofibroblast-Derived Exosome Induce Cardiac Endothelial Cell Dysfunction
Source: Front Cardiovasc Med. 2021 Apr 23;8:676267. doi: 10.3389/fcvm.2021.676267 (PMC8102743; doi:10.3389/fcvm.2021.676267)
Supplement: Supplementary file 2 [file Table_2.DOCX]

*List of Antibodies used*

| **S. No.** | **Antibodies (Cell Signaling Technology)** | **Catalog Number** |
| --- | --- | --- |
| 1. | Phospho-P38 | 4511S |
| 2. | P-38 | 9228S |
| 3. | Phospho SMAD2 | 18338S |
| 4. | SMAD2/3 | 8685S |
| 5. | GAPDH | 5174s |
| 6. | Β-Actin | 3700S |
| 7. | Hif1α | 14179S |
| 8. | Bcl2 | 3498S |
| 9. | Cleaved Caspase 3 | 9664S |
| 10. | ETS1 | 14069S |
| 11. | CD31 | 3528S |
| 12. | eNOS | 32027S |
| 13. | α-SMA | 19245S |
| 14. | Anti-Mouse Igg, Hrp-Linked Antibody | 7076S |
| 15. | Anti-Rat Igg, Hrp-Linked Antibody | 7077 |
| **S. No.** | **Antibodies (Abcam)** | **Catalog Number** |
| 16. | Fibronectin | Ab32419 |
| 17. | PIGF | Ab196666 |
| 18. | VEGF-A | Ab46154 |
| **S. No.** | **Antibodies (Invitrogen)** | **Catalog Number** |
| 19. | Phospho-eNOS | MA5-14957 |

## Supplementary Table S2. *. We have enlisted here the details of all antibodies used in this work.*
