# Supplementary material for: Myofibroblast-Derived Exosome Induce Cardiac Endothelial Cell Dysfunction
Source: Front Cardiovasc Med. 2021 Apr 23;8:676267. doi: 10.3389/fcvm.2021.676267 (PMC8102743; doi:10.3389/fcvm.2021.676267)
Supplement: Supplementary file 3 [file Data_Sheet_1.DOCX]

TGFβ treatment activates adult mice cardiac fibroblasts.


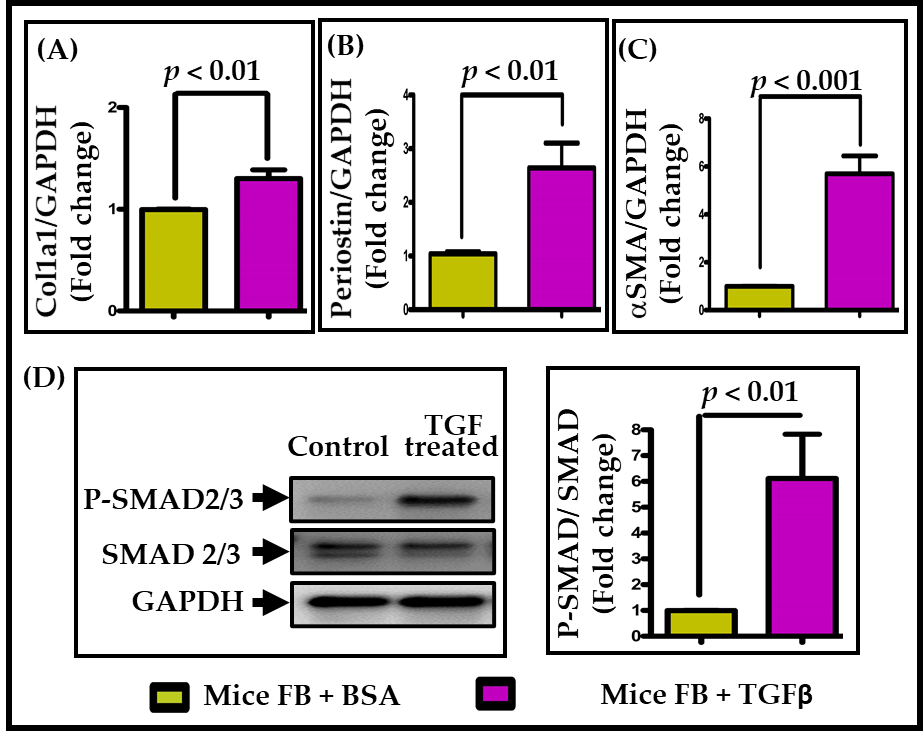


## Supplementary Figure S1. TGFβ treatment activates adult mice cardiac fibroblasts. TGFβ treatment significantly upregulated pro-fibrotic genes, including (A) Col1a1, (B) Periostin and (C) αSMA in mice cardiac fibroblast cells. In addition, an increased (D) phosphorylation of SMAD2/3 was observed with activation of mice cardiac fibroblast by TGFβ. Densitometry data of western blot showing phosphorylated vs total protein. GAPDH was used as loading control for both gene and protein expression analysis. p<0.05 was considered as statistical significance.
