# Supplementary material for: Myofibroblast-Derived Exosome Induce Cardiac Endothelial Cell Dysfunction
Source: Front Cardiovasc Med. 2021 Apr 23;8:676267. doi: 10.3389/fcvm.2021.676267 (PMC8102743; doi:10.3389/fcvm.2021.676267)
Supplement: Supplementary file 4 [file Data_Sheet_2.DOCX]

Number of exosomes.

**
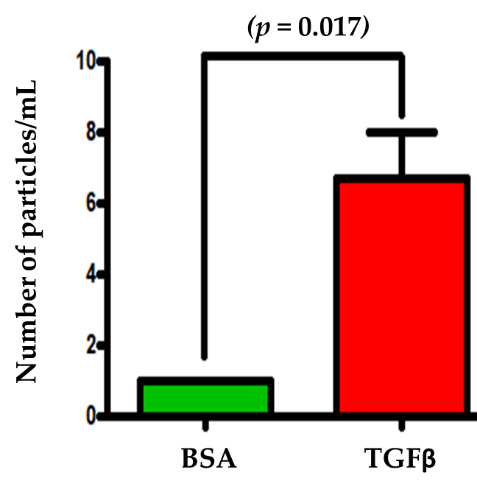
**

## Supplementary Figure S2. Number of exosomes. The number of exosomes per mL was significantly higher in TGFβ treated fibroblast cells. However, their sizes were in similar range (50-130 nm) with peak at around 100nm from both the treatments.
