# Supplementary material for: Myofibroblast-Derived Exosome Induce Cardiac Endothelial Cell Dysfunction
Source: Front Cardiovasc Med. 2021 Apr 23;8:676267. doi: 10.3389/fcvm.2021.676267 (PMC8102743; doi:10.3389/fcvm.2021.676267)
Supplement: Supplementary file 5 [file Data_Sheet_3.DOCX]

*Mouse Fibrosis miScript miRNA array along with expression profile and validation of selected miRNAs in exosomes.*


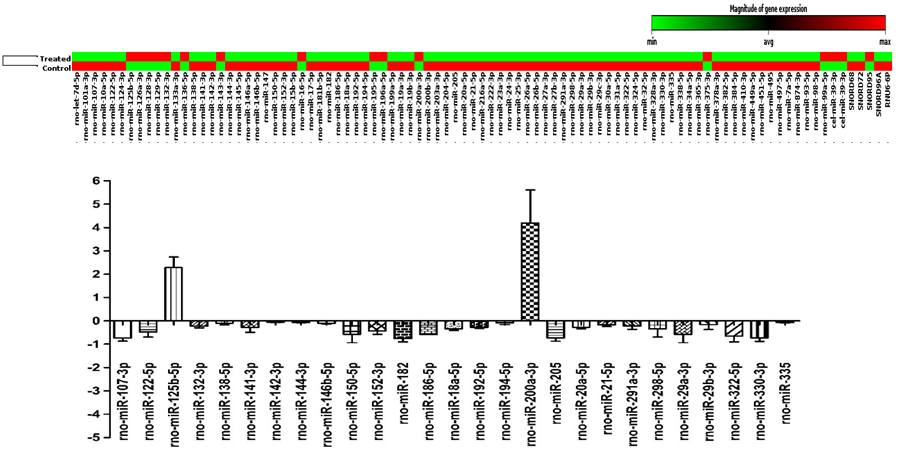


## Supplementary Figure S3. Mouse Fibrosis miScript miRNA array along with expression profile and validation of selected miRNAs in exosomes. Cardiac fibroblast cells were isolated from adult mice heart. Fibrosis-miRNAs array was performed in cardiac fibroblast-derived exosomes after TGFβ activation. The fibrotic miRNAs array data of mice showed similar result as in rat.
