# Supplementary material for: Myofibroblast-Derived Exosome Induce Cardiac Endothelial Cell Dysfunction
Source: Front Cardiovasc Med. 2021 Apr 23;8:676267. doi: 10.3389/fcvm.2021.676267 (PMC8102743; doi:10.3389/fcvm.2021.676267)
Supplement: Supplementary file 6 [file Data_Sheet_4.DOCX]

*Dot blot analysis using Proteome Profiler™ Mouse Angiogenesis Antibody Array (R&D Systems).*

**
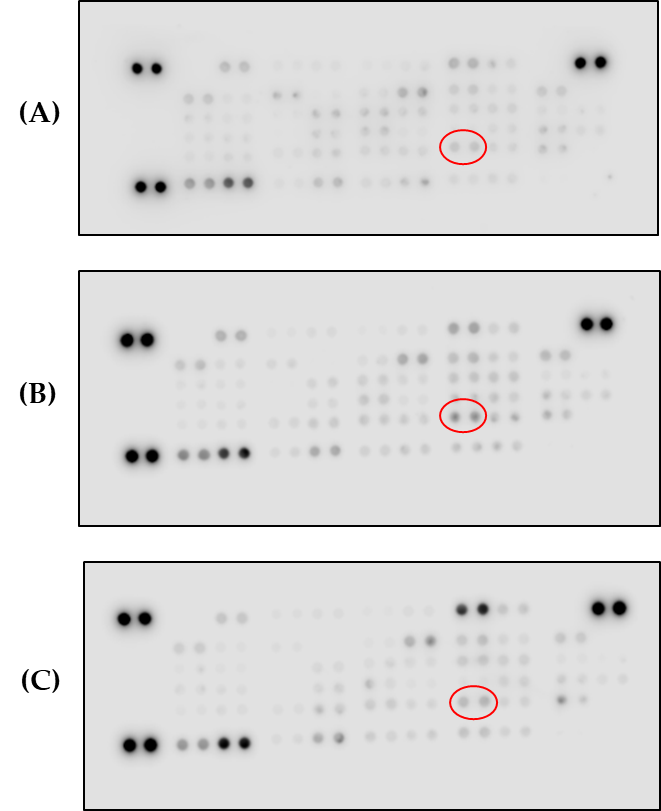
**

## Supplementary Figure S4. Dot blot analysis using Proteome Profiler™ Mouse Angiogenesis Antibody Array (R&D Systems). Analysis of angiogenesis-related proteins showed that PIGF expression was greatly reduced in endothelial cells treated with FB-TGFβ-Exosomes (C) as compared to PBS and control exosomes treatment (A, B) when normalized with reference controls.
